# Covid-19 als Chance für den Frieden? Ernüchternde Erfahrungen aus Kolumbien und Syrien

**DOI:** 10.1007/s42597-020-00044-y

**Published:** 2020-11-03

**Authors:** André Bank, Sabine Kurtenbach

**Affiliations:** Leibniz-Institut für Globale und Regionale Studien, Neuer Jungfernstieg 21, 20354 Hamburg, Deutschland

**Keywords:** Corona, Pandemie, Gewalt, Waffenstillstand, Nichtstaatliche Gewaltakteure, Corona, Pandemic, Violence, Ceasefire, Nonstate armed actors

## Abstract

Ende März 2020 rief UN-Generalsekretär Guterres angesichts der Bedrohung durch die Covid-19-Pandemie dazu auf, weltweit die Waffen ruhen zu lassen. Obwohl diese Initiative von 171 Regierungen begrüßt wurde, ist es nicht einmal kurzfristig zu einer umfassenden Gewaltreduktion in zentralen Konfliktgebieten gekommen. Der vorliegende Beitrag untersucht die Auswirkungen der Corona-Pandemie in zwei regional wie global bedeutsamen Konfliktländern, Kolumbien mit seiner fragilen Befriedung und Syrien mit einem militärisch fast entschiedenen Krieg. Er fragt, wie die Corona-Krise die Dynamiken von Gewalt und Frieden nach bzw. am Ende von Bürgerkriegen beeinflusst.

Kolumbien gilt weltweit als Beispiel dafür, dass umfassende Friedensabkommen auch in komplexen Konflikten möglich sind. Trotzdem ist der kurzfristige, friedenspolitische Trend in Zeiten der Pandemie bestenfalls ambivalent: Zwar hat die absolute Zahl der Morde abgenommen, MenschenrechtsverteidigerInnen und demobilisierte Ex-KombattantInnen bleiben jedoch bevorzugte Gewaltopfer, die Zahl der Massaker hat wieder zugenommen. Die noch aktive Guerillagruppe Ejército de Liberación Nacional (ELN) verkündete zunächst einen einseitigen Waffenstillstand, beendete diesen aber Ende April. Auch in Syrien sind die Auswirkungen der Pandemie auf die Konfliktdynamik widersprüchlich bis negativ: In der letzten Rebellenhochburg Idlib hat Covid-19 zwar indirekt dazu beigetragen, die russisch-türkische Waffenruhe vom März 2020 über mehrere Monate zu stabilisieren. Im Nordosten Syriens, der teils von der Türkei, teils von den kurdisch dominierten Syrian Democratic Forces (SDF) kontrolliert wird, hat sich die humanitäre Lage hingegen seit Beginn der Pandemie deutlich verschlechtert, da das Assad-Regime die internationale Hilfe nicht in das verfeindete Gebiet weiterleitet. Trotz diverser Unterschiede zwischen Kolumbien und Syrien nutzen die jeweiligen Regierungen die mit der Ausbreitung des Virus verbundenen Krisen vor Ort, um ihre politischen Agenden gewaltsam zu festigen. In einem größerem Kontext zeigen die Erfahrungen in beiden Ländern, dass Covid-19 lokal weniger als „game changer“ denn als Verstärker und Beschleuniger von Dynamiken wirkt, die bereits vor Ausbruch der Pandemie bestanden hatten.

## Abstract (en)

Given the threat of the COVID-19 pandemic, UN General Secretary Guterres called for ceasefires around the globe in late March 2020. Even though this initiative was welcomed by 171 governments, not even a short-term violence reduction has happened in central conflict areas. This contribution examines the effects of the Corona pandemic in two regionally and globally significant conflict countries, Colombia with its fragile pacification and Syria with its militarily almost decided war. The article asks how the Corona crisis influences the dynamics of violence and peace after respectively at the end of civil wars. Colombia is a prominent example of a comprehensive peace agreement in complex conflicts. Nonetheless, the short-term, peace-related trend in times of the pandemic is ambivalent at best: While the absolute number of murders has declined, it is human right defenders and demobilized ex-combatants who remain the main victims of violence - and the number of massacres has increased again. The still-active guerilla Ejército de Liberación Nacional (ELN) initially called for a one-sided truce, yet ended again in late April. In Syria, the consequences of the pandemic on the conflict dynamic have ranged from contradictory to negative: In the last rebel stronghold Idlib COVID-19 directly contributed to the months-long stabilization of the Russian-Turkish truce from March 2020. In Northeastern Syria, which is partly controlled by Turkey and partly by the Kurdish-dominated Syrian Democratic Forces (SDF), the humanitarian situation has clearly worsened since the beginning of the pandemic. The Assad regime does not allow international aid to pass through to the opposition territory. Despite diverse differences between Colombia and Syria, one crucial similarity is that the respective governments instrumentalize the crisis on the ground for the often violent consolidation of the political agenda. In the bigger picture, the experience from the two countries suggests that COVID-19 works locally less as a “game-changer”, but rather as intensifier or accelerator of pre-pandemic dynamics.

## Einleitung: Covid-19 und die Auswirkungen auf Gewalt und Frieden

Die Covid-19-Pandemie betrifft alle Länder rund um den Globus. Staaten und Gesellschaften stehen nicht nur medizinischen und gesundheitsbezogenen Herausforderungen gegenüber, sondern müssen sich zugleich mit den gravierenden ökonomischen und politischen Konsequenzen der Pandemie auseinandersetzen. In Bürgerkriegs- und Nachkriegsländern besteht außerdem die Gefahr, dass die Pandemie innergesellschaftliche Konflikte verschärfen, zu neuerlicher Gewalt beitragen und somit noch mehr Menschenleben kosten könnte. Vor diesem Hintergrund rief António Guterres, der Generalsekretär der Vereinten Nationen (UN), am 23. März und 6. April 2020 eindringlich dazu auf, angesichts der Bedrohung durch Covid-19 in Gewaltkonflikten weltweit die Waffen ruhen zu lassen (UN 2020a). Seine Initiative eines globalen Waffenstillstands wurde bis Ende Juni 2020 von 171 Regierungen unterstützt. Es dauerte jedoch über drei Monate, bis der UN-Sicherheitsrat am 1. Juli 2020 die Resolution 2532 verabschiedete, die dafür plädiert, dass zumindest für 90 Tage alle Konfliktakteure eine humanitäre Pause der Kämpfe einhalten sollen (UN 2020b).

Die Appelle des UN-Generalsekretärs sowie die Resolution 2532 verweisen auf die möglichen negativen Auswirkungen von Covid-19 auf Frieden und Sicherheit. Empirisch bleibt bis dato jedoch offen, ob die mit der Ausbreitung des Virus verbundenen Krisen vor Ort tatsächlich die Gewalt anheizen, ob sie unter spezifischen Umständen gar einer Befriedung dienlich sind oder ob sie keinen oder nur sehr begrenzt Einfluss auf die jeweilige Konfliktdynamik haben. Der vorliegende Beitrag analysiert den kurzfristigen Einfluss der Corona-Krise vergleichend in zwei dieser „gefährlichen“ Kontexte: Kolumbien als ein Beispiel fragiler Befriedung sowie Syrien als ein Fall eines militärisch fast entschiedenen Krieges. Trotz grundlegender Unterschiede hinsichtlich Gewaltakteuren, -dynamik und -ökonomie teilen Kolumbien und Syrien die Erfahrung langjähriger, extrem gewaltsamer Bürgerkriege mit großen subnationalen Unterschieden sowie die grenzüberschreitende Bedeutung der Konflikte für ihre jeweiligen Regionen Lateinamerika bzw. den Nahen Osten.

Syrien war eines der ersten Länder, die Guterres in seinem globalen Appell nannte. Dies verwundert nicht, ist es doch das Land mit dem brutalsten Bürgerkrieg des 21. Jahrhunderts: Für März 2020, neun Jahre nach Beginn des Gewaltkonflikts, zählte die oppositionsnahe Syrische Beobachtungsstelle für Menschenrechte zwischen 384.000 und 586.100 Todesopfer (SOHR 2020). Die Zahl der Verletzten ist noch höher und über elf Millionen SyrerInnen sind entweder intern vertrieben oder ins Ausland geflohen. In jüngerer Zeit zeichnet sich in Syrien ein militärischer „Siegfrieden“ ab, seit das diktatorische Regime unter Präsident Bashar al-Assad, zusammen mit seinen Verbündeten Russland und Iran, ungefähr zwei Drittel des Territoriums kontrolliert. Es war in dieser Phase des Konflikts, am 22. März 2020, als die syrische Regierung den ersten Fall von Covid-19 in Syrien offiziell bestätigte. Dies war eine relativ späte Bekanntgabe, da es in den Nachbarländern Iran, Irak und Libanon bereits deutlich früher Krankheitsausbrüche gegeben hatte (19. bzw. 22. Februar) und Syrien gerade zu Iran – dem anfänglichen Epizentrum von Covid-19 im Nahen Osten – besonders enge personelle Verbindungen durch reisende Militärs, Geschäftsleute und schiitische PilgerInnen hatte. Ein halbes Jahr später, am 17. September 2020, gab das syrische Gesundheitsministerium die Zahl offiziell bestätigter Fälle von Covid-19 in Syrien mit 3654 an, 163 Menschen sollen gestorben sein (Syrian Ministry of Health 2020). Diese sehr niedrigen Infektions- und Todeszahlen sollten angesichts fehlender Testkapazitäten im Land sowie der Tendenz des syrischen Regimes, Statistiken fälschlich darzustellen, mit großer Vorsicht betrachtet werden. Nichtsdestotrotz kann davon ausgegangen werden, dass es in Syrien bis zum Sommer 2020 nicht zu einem massiven Ausbruch von Covid-19 gekommen ist.

In Kolumbien wurde der erste Covid-19-Fall am 6. März 2020 registriert, bis zum 17. September meldete das Gesundheitsministerium 736.377 Infektionen und 23.478 Tote (Ministerio de Salud 2020). Auch wenn die Hauptstadt Bogotá mit ihren über zehn Millionen EinwohnerInnen am stärksten betroffen ist, hat sich das Virus mittlerweile über das gesamte Land ausgebreitet. Die Regierung hatte von 23. März 2020 bis Ende August eine umfassende Quarantäne verhängt, die aber nur begrenzt eingehalten wurde. Vor allem für die fast 50 % informell Beschäftigten wiegt die Ansteckungsgefahr geringer als die Notwendigkeit, den Unterhalt für das tagtägliche Überleben zu sichern. Die Pandemie trifft Kolumbien zu einem politisch sehr schwierigen Zeitpunkt: Knapp vier Jahre nach Unterzeichnung des weltweit umfassendsten Friedensabkommens (6 Kapitel, 578 Artikel) bleibt das Land tief gespalten und polarisiert. Die Regierung Duque, seit 2018 im Amt, hat die Umsetzung nicht nur verschleppt, sondern versucht, zentrale Teile wie das Abkommen zur Übergangsjustiz auszuhebeln. Noch befinden sich im parteipolitisch fragmentierten Parlament diejenigen in der Mehrheit, die das Abkommen im Wesentlichen unterstützen. Bei den Kommunalwahlen Mitte 2019 haben sich in allen großen und einigen mittleren Städten reformorientierte Koalitionen durchgesetzt, die auf eine substanzielle Umsetzung des Abkommens drängen. Die Gewalt hat im letzten Jahrzehnt insgesamt abgenommen, allerdings bleiben zahlreiche bewaffnete Gruppen aktiv. Dazu gehören das Ejército Nacional de Liberación (ELN) als letzte Guerillagruppe, die seit Mitte der 1960er Jahre für ein anderes Entwicklungsmodell kämpft. Knapp 1000 ehemalige KämpferInnen der Fuerzas Armadas Revolucionarias de Colombia-Ejército del Pueblo (FARC-EP) haben sich entweder nicht demobilisiert oder sind erneut in den Untergrund abgetaucht. Darüber hinaus sind sogenannte kriminelle Banden aktiv, die teilweise transnational in der illegalen Ökonomie agieren (Ávila 2019). Diese politische Fragmentierung, die anhaltende politische Gewalt und die darunter liegenden Konflikte erschweren einen kohärenten Umgang mit der Pandemie in Kolumbien.

Unser Forumsbeitrag ist wie folgt strukturiert: Der nächste Teil behandelt die theoretische Debatte, unter welchen Bedingungen Pandemien friedensstiftend oder gewaltfördernd sind. Daran anschließend wird anhand aktueller Waffenstillstände in Kolumbien und Syrien eruiert, ob und wenn ja, wie sich friedensfördernde Kooperationen im Kontext von Covid-19 entwickeln können. Der vierte Teil konzentriert sich auf die fragmentierte Territorialkontrolle in beiden Ländern und nimmt dabei auch die Rolle nichtstaatlicher Gewaltakteure in den Blick. Abschließend wird vor dem Hintergrund der Erfahrungen Kolumbiens und Syriens diskutiert, wie Covid-19 auf Frieden und Gewalt wirkt.

## Pandemien als Friedensstifter oder Brandbeschleuniger?

Die Covid-19-Pandemie ist zwar ein globales Phänomen, ihre Folgen sind allerdings sehr unterschiedlich. In armen, fragilen und gewaltgeprägten Ländern werden große Verwerfungen erwartet, weil die gesellschaftlichen und wirtschaftlichen Auswirkungen von Quarantäne und sozialer Distanzierung das (Über‑)Leben großer Bevölkerungsgruppen gefährden. Vor diesem Hintergrund warnen viele Berichte vor einer einsetzenden Welle neuer oder der Verschärfung bestehender Gewaltkonflikte (IEP 2020). Einen anderen Ansatz verfolgt – wie eingangs erwähnt – UN-Generalsekretär Guterres, der mit seinem Plädoyer für einen zumindest temporären Waffenstillstand staatlicher wie nichtstaatlicher Gewaltakteure in Konfliktländern rund um den Globus nicht nur eine effektivere Bekämpfung von Covid-19 ermöglichen möchte. Vielmehr, so die Hoffnung, könnten sich durch Kooperation gegen Covid-19 eine Annäherung der GegnerInnen und im Idealfall auch neue Räume für diplomatische Initiativen der Konfliktbearbeitung jenseits der Pandemiebekämpfung ergeben.

Theoretisch lassen sich die Auswirkungen von Pandemien auf Gewalt oder Frieden unter zwei Perspektiven analysieren: Erstens lassen sich Pandemien als Chance für verstärkte Kooperation betrachten, die zu einem Rückgang von Gewalt und zu mehr Frieden beitragen können. Dieser Ansatz geht davon aus, dass die eher technische Kooperation zur Bewältigung einer gemeinsamen Bedrohung Vertrauen schafft. Auf dieser Basis können dann kontroversere politische Probleme leichter angegangen werden. In einer Studie zur Kooperation zwischen Israel, Jordanien und der palästinensischen Autonomiebehörde während der Vogelgrippe 2006 zeigt William J. Long (2011) allerdings, dass die Zusammenarbeit nur sehr punktuell war und es zu einem schnellen „Rückfall“ in altbekannte Konfliktmuster kam. Die Weltgesundheitsorganisation (WHO) propagiert mit ihrem Programm *Health as a Bridge to Peace* einen ähnlichen Ansatz auf der lokalen Ebene. Im Kontext von Naturkatastrophen gibt es eine vergleichbare Diskussion, aber auch hier ist die empirische Evidenz des positiven Zusammenhangs von externem Schock und Frieden lediglich begrenzt: Im häufig genannten Beispiel Aceh/Indonesien befand sich der Friedensprozess bereits vor dem Tsunami Ende Dezember 2004 in Vorbereitung; die Katastrophe beschleunigte die Finalisierung des Friedensvertrags also bestenfalls (Gaillard et al. 2008). Die Auswirkungen von externen Schocks, seien es Pandemien oder Naturkatastrophen, sind folglich ambivalent und in hohem Maße kontextabhängig.

Eine zweite Perspektive ergibt sich mit Blick auf die Exekutive, der auf unterschiedlichen Ebenen (national, subnational, lokal) in Krisenzeiten eine besondere Rolle zukommt. Die etwa von der WHO empfohlenen Maßnahmen der sozialen Distanzierung, Lockdown und Quarantäne schränken grundlegende individuelle und kollektive Rechte ein. Die Durchsetzung dieser Freiheitsbeschränkungen ruft deshalb nicht selten neue Konflikte und Proteste hervor. Das Datenprojekt ACLED dokumentiert für die vier Monate zwischen der Erklärung von Covid-19 zur Pandemie durch die WHO am 11. März und dem 11. Juli 2020, wie sich unterschiedliche Formen der Gewalt im Kontext der Pandemie verändert haben. Dem in globaler Betrachtung leichten Rückgang politischer Gewalt stehen sehr vielfältige Entwicklungen in den Regionen und bei verschiedenen Formen der Gewalt gegenüber (Pavlik 2020). Gelungenes oder gescheitertes Krisenmanagement in Zeiten von Pandemien kann die Zustimmung zur jeweiligen Regierung entweder erhöhen oder aber ins Bodenlose sinken lassen (Tsai et al. 2020). Dies gilt auch im Kontext aktueller wie vormaliger Gewaltkonflikte, wo die entsprechenden Maßnahmen unter Umständen nicht vom Staat, sondern von nichtstaatlichen bewaffneten Akteuren durchgesetzt werden.

Weder bei der Perspektive auf Kooperation noch bei der auf die Exekutiven ergibt es allerdings Sinn, die Analyse auf die Makroebene nationaler Staaten zu beschränken. Die Friedens- und Konfliktforschung hat zu Recht lokale und subnationale Dynamiken von Gewalt und Frieden in den Fokus der Betrachtung gerückt (Björkdahl und Buckley-Zistel 2016). Entwicklungen auf der subnationalen Ebene beeinflussen Krieg und Frieden in vielerlei Hinsicht. Auch lokal begrenzte Gewalt kann fragile Friedensprozesse schwer belasten, wenn sie politisch instrumentalisiert wird, Repression rechtfertigt und zu erneuter Flucht und Vertreibung führt. Die Einbeziehung unterschiedlicher subnationaler Entwicklungen erlaubt es zudem, innerhalb eines Landes die komplexen Beziehungen zwischen lokalen und nationalen Eliten zu betrachten. Die Entstehung und Dynamik transnationaler Konflikte ergibt sich oftmals über die lokalen Ebenen in Grenzregionen. Der Vergleich der Entwicklungen in Kolumbien und Syrien unter diesen beiden Gesichtspunkten und in unterschiedlichen subnationalen Räumen zeigt, wie sich Covid-19 auf verschiedene Formen der Gewalt und die Potenziale von Frieden auswirkt.

## Waffenstillstände in Kolumbien und Syrien: Begrenzte Effekte von Covid-19

Die in Kolumbien nach dem Friedensabkommen von 2016 weiterhin aktive Guerillagruppe ELN entstand in den 1960er Jahren im Umfeld studentischer Organisationen und der Befreiungstheologie. In der Tradition des kubanischen Befreiungskampfes sollte die Landbevölkerung mobilisiert werden. Politisch tritt das ELN für eine Nationalisierung der Rohstoffe, insbesondere des Erdöls, ein; die Zahl ihrer KämpferInnen betrug in der Hochphase circa 2000. Die Finanzierung des ELN erfolgt vor allem durch Entführungen, Schutzgelderpressungen und Drogenhandel. Nach dem Friedensabkommen zwischen der Regierung Santos und der FARC-EP 2016 erhöhte sich der politische Druck sowohl auf das ELN als auch auf die Regierung, auch den ELN-bezogenen Gewaltkonflikt zu beenden. Nach dreimonatigen Vorgesprächen begannen im Februar 2017 formelle Verhandlungen in Havanna. Sie verliefen zäh und kamen mit dem Regierungswechsel zum Rechtspolitiker Duque im August 2018 zum Stillstand. Das Attentat des ELN auf eine Polizeischule in Bogotá im Januar 2019, bei dem 22 Menschen starben, bedeutete sodann zunächst das Aus für die Gespräche.

Dem Aufruf des UN-Generalsekretärs zum Waffenstillstand folgte das ELN Anfang April 2020 mit der Erklärung einer einseitigen, einmonatigen Waffenruhe aus einer Position der Schwäche. Ziel war es, die Regierung Duque zur Rückkehr an den Verhandlungstisch zu bewegen. Während die Regierung militärisch den Druck in den Anfangsmonaten 2020 deutlich erhöhte, initiierte sie zugleich ein Programm, dass die individuelle Demobilisierung von ELN und FARC-Dissidenten fördern sollte (Llorente 2020). Auch internationale Haftbefehle gegen die ELN-Unterhändler, die sich noch in Havanna befanden, waren ein deutliches Signal, dass die Regierung keinerlei Interesse an Gesprächen hat, sondern auf einen militärischen Sieg setzt. Ende Juni 2020 – inmitten des allgemeinen „Lockdowns“ in Kolumbien – verhafteten Sicherheitskräfte zudem acht mutmaßlich für den 2019er Anschlag verantwortliche ELN-Mitglieder. Auch auf den neuerlichen Aufruf des ELN zu einem 90-tägigen Waffenstillstand Anfang Juli 2020 ging die Regierung nicht ein, sondern forderte vom ELN die Niederlegung der Waffen ohne Zugeständnisse. Im kolumbianischen Fall ermöglichte die Ausbreitung der Covid-19-Pandemie somit bis dato kein „window of opportunity“ für einen neuerlichen Waffenstillstand, weil übergreifende Macht- und Sicherheitsinteressen der Regierung Duque klar dominierten.

Im Gegensatz zu Kolumbien zeichnet sich in Syrien ein brutaler „Siegfrieden“ des Assad-Regimes und seiner externen Verbündeten ab. Es war vor allem Russlands Militärintervention seit September 2015, die die Wiedereroberung der nordsyrischen Metropole Aleppo 2016/17 ermöglichte. Mit Hilfe der russischen Luftwaffe, Iran-finanzierter Milizen und der libanesischen Hizballah gelang es den Regimekräften während der Jahre 2017/18, die vormaligen Rebellenhochburgen Dar’a, Ost-Ghouta sowie das Umland von Homs und Hama gewaltsam unter Kontrolle zu bringen. Auf der Grundlage sogenannter „Versöhnungsabkommen“, die von Assad und seinen Verbündeten oktroyiert wurden und de facto „Knebelverträge“ sind (Sosnowski 2019), flohen die in den vormaligen Oppositionsgebieten lebenden KämpferInnen sowie große Teile der dortigen Zivilbevölkerung in die letzte verbliebene Rebellenhochburg Idlib im Nordwesten Syriens.

Seither leben ungefähr drei Millionen Menschen in Idlib, circa 1,5 Mio. von ihnen sind Binnenflüchtlinge. Während sich die humanitäre Lage vor Ort verschlechterte, verhinderte das russisch-türkische Sotschi-Abkommen vom September 2018 zunächst eine Großoffensive des syrischen Regimes auf das Gebiet. Nach dem graduellen Niedergang des Sotschi-Abkommens begann Ende April 2019 die Offensive der syrischen und russischen Luftwaffen auf Idlib. Bis Ende Februar 2020 führten die Kämpfe abermals zum Tod von Tausenden ZivilistInnen und zu internen Fluchtbewegungen. Ein Luftangriff auf eine türkische Kommandozentrale am 27. Februar 2020 tötete mindestens 33 SoldatInnen. Alarmiert durch die Gefahr einer noch massiveren Gewalteskalation begannen Russland und die Türkei umgehend und unter Ausschluss Assads Verhandlungen, die am 5. März in einen bilateralen Waffenstillstand für Idlib mündeten. Erst im Juli 2020 kam es wieder zu vereinzelten Kampfhandlungen in Idlib, die jedoch bei weitem nicht das Gewaltniveau vor März 2020 erreichten.

Welcher Einfluss kommt der Covid-19-Pandemie angesichts des mehrmonatigen Fortbestehens des russisch-türkischen Waffenstillstands in Idlib zu? Die kurze Antwort lautet: Ein begrenzter und höchstens indirekter Einfluss. Für Russland unter Putin wie für die Türkei unter Erdoğan waren bei der Initiierung wie bei der Aufrechterhaltung der bilateralen Waffenruhe primär geostrategisch-sicherheitspolitische und ökonomische, jedoch keine direkt Pandemie-bezogenen Erwägungen maßgeblich (Bank 2020). Soweit es nicht zu massiven anti-russischen Operationen durch die radikal-islamistische, jihadistische Rebellenorganisation HTS („Hai’at Tahrir ash-Sham“, Komitee zur Befreiung Großsyriens), die auf der lokalen Ebene in Idlib herrscht, oder einem noch umfassenderen Truppenausbau der Türkei in dem Gebiet kommt, ist Russland wenig an einer größeren Gewalteskalation in Idlib gelegen. Die Türkei hat ihrerseits ebenfalls ein Interesse an der Aufrechterhaltung der Waffenruhe vom 5. März. Aus Ankaras Sicht verringert sie den Druck der Binnenflüchtlinge, die zuvor angesichts der heftigen Bombardements immer wieder auf der Flucht in Richtung türkische Grenze waren. Innerhalb Idlibs konnte die Türkei zudem ihre militärischen Positionen nach dem Ende der Kämpfe weiter ausbauen.

Während Covid-19 in den ersten Monaten weder für Russland noch für die Türkei einen nennenswerten Einfluss auf die jeweiligen Syrien-Strategien sowie, spezifischer, den Waffenstillstand in Idlib zu haben schien, so könnte sich dies zukünftig ändern. Maßgeblich hierfür wäre erstens ein massiver Ausbruch der Pandemie in Syrien selbst, der auch türkische und russische Truppen im Nordwesten gefährden könnte. Offiziell wurde Anfang Juli 2020 der erste Covid-19-Fall in Idlib festgestellt. Angesichts der sehr unsicheren Datenlage zu Nordwestsyrien, die vom fehlenden Zugang von monitoring-Organisationen wie den UN und NGOs erschwert wird, liegen lediglich Einzelinformationen von Hilfsorganisationen vor, die von einer raschen Ausbreitung der Pandemie beim Gesundheitspersonal seit Juli 2020 berichten (Ärzte ohne Grenzen 2020) bzw. unterstreichen, dass es bis Mitte Juli lediglich 10.000 Tests für Gesamtsyrien gegeben haben soll. Zweitens dürfte sich in naher Zukunft deutlicher bemerkbar machen, dass die Pandemie „zu Hause“ in Russland und der Türkei so massiv negative gesundheitsbezogene wie ökonomische Auswirkungen hat, dass die jeweiligen außen- und sicherheitspolitischen Gestaltungsspielräume in Syrien klarer eingeschränkt wären.

## Fragmentierte Territorialkontrolle und (nicht-)staatliche Gewaltakteure in Zeiten von Covid-19

Angesichts ihrer langen Gewaltgeschichte werden Syrien als Kriegsland sowie Kolumbien als Nachkriegsland bis heute von einer Vielzahl staatlicher wie nichtstaatlicher Gewaltakteure dominiert. In beiden Ländern stellen dabei die staatlichen Sicherheitskräfte einflussreiche, aber nicht notwendigerweise immer die mächtigsten und angesichts massivster staatlicher Menschenrechtsverletzungen schon gar nicht immer die legitimsten Gewaltakteure dar. Eng mit der Vielfalt von Gewaltakteuren verbunden ist die fortbestehende Fragmentierung der Territorialkontrolle in Syrien und Kolumbien; in beiden Ländern kommt hierbei sogenannten peripheren Gebieten jenseits der jeweiligen Hauptstädte eine zentrale Bedeutung zu.

In Syrien stellt der Nordosten neben der nordwestlichen Region Idlib das zweite, subnational und lokal umkämpfte Gebiet des Landes dar. Traditionell lebten in der Region östlich des Euphrat-Flusses vornehmlich arabische und kurdische Großfamilien, oft mit grenzüberschreitenden Verbindungen in den Irak und die Türkei. In der Anfangsphase des Syrienkriegs 2012 gelang es insbesondere den kurdischen Volksverteidigungseinheiten („Yekîneyên Parastina Gel“, YPG) ein größeres Territorium infolge des Rückzugs der Regimekräfte militärisch unter Kontrolle bringen. Dieses von den syrischen KurdInnen als „Rojava“ (Westkurdistan) bezeichnete Gebiet war seither Angriffen höchst unterschiedlicher innersyrischer, nahöstlicher wie außerregionaler Gewaltakteure ausgesetzt: Die Anti-Regime-Rebellen der primär aus desertierten syrischen Soldaten bestehenden, von der Türkei unterstützten „Freien Syrischen Armee“ (FSA) kämpften dabei gegen die YPG um Gebiete im Umland Aleppos. Der radikal-islamistische Islamische Staat (IS) trat seit 2013 aus Westirak kommend massiv im Osten Syriens auf; er kontrollierte zeitweise einen Großteil Nordostsyriens und diverse Gebiete darüber hinaus. Unterstützt durch die internationale Allianz gegen den IS, in der den USA eine herausgehobene Bedeutung zukam, bekämpften die YPG und die ethnisch-religiös pluralistischeren SDF („Syrian Democratic Forces“) sehr verlustreich, aber letztlich erfolgreich den IS. Des Weiteren intervenierte das anti-kurdisch ausgerichtete türkische Militär mehrfach in der Region östlich des Euphrats, zusätzlich auch syrische Regimekräfte, Russland und mit Iran verbundene Milizen.

Die Ankündigung von US-Präsident Trump vom 7. Oktober 2019, dass sich die US-Truppen komplett aus Syrien zurückziehen würden, ermöglichte eine neuerliche Invasion des türkischen Militärs in Nordostsyrien. Ankaras Bodentruppen vertrieben die kurdischen YPG, die seit dem Kampf gegen den IS unter der Protektion der US-Truppen in Syrien gestanden hatte. Als Washington verkündete, dass es doch ein Kontingent von ungefähr 400 US-SoldatInnen in Syrien belassen würde, kam Russland mit der Türkei Ende Oktober 2019 überein, eine circa 100 km lange „Sicherheitszone“ an der syrisch-türkischen Grenze zu errichten. Das syrische Regime entsandte Truppen und auch von Iran unterstützten Fatemiyoun- und Zaynabiyoun-Brigaden operieren im syrisch-irakischen Grenzland. Zusammen genommen entwickelte sich so eine komplexe Form der fragmentierten Territorialkontrolle zwischen der kurdischen YPG, den kurdisch dominierten SDF, Teheran-alliierten Milizen, der syrischen Armee sowie den Truppen Russland, der Türkei und den USA.

Für die Zivilbevölkerung war und ist in diesem Kontext die überlebenswichtige Versorgung mit Lebensmitteln und Medikamenten eine immense Herausforderung. Mit der Schließung des wichtigen syrisch-irakischen Grenzpostens Yarubiyah im Januar 2020 durch Russland wurde die Zivilbevölkerung von den wenigen humanitären Hilfslieferungen der UN abgeschnitten und ihre Versorgung mit Lebensmitteln und Medikamenten fast vollständig in die Hände des Assad-Regimes gelegt. Gerade im Kontext der beginnenden Covid-19-Pandemie mit den strikteren Grenzschließungen und selektiven Ausgangssperren seit dem Frühjahr 2020 gab dies dem syrischen Regime einen wichtigen Hebel, vermeintliche Loyalität der Menschen im Nordostsyrien via Hilfslieferungen zu honorieren und oder aber vermeintliche Illoyalität durch die Nichtweiterleitung lebensnotwendiger Güter zu bestrafen. Diese Lage wurde durch die Resolution 2533 des UN-Sicherheitsrats vom 11. Juli 2020 nochmals bestätigt, da der Grenzübergang Yarubiyah für internationale Hilfslieferungen für mindestens zwölf Monate geschlossen bleibt (UN 2020c). Im Spätsommer 2020 gab es im gesamten Nordosten Syriens ein Spezialkrankenhaus für Covid-19, das in der Nähe der Stadt Hassakeh liegt, gleichzeitig jedoch steigende Infektionszahlen, unter anderen in Hassakeh und Qamishli (Ärzte ohne Grenzen 2020). Vor diesem Hintergrund lässt sich festhalten, dass die fragmentierte Territorialkontrolle multipler Gewaltakteure bereits vor 2020 die Situation für die Zivilbevölkerung vor Ort massiv erschwert hat. Der Ausbruch von Covid-19 in Syriens Nordosten seit Anfang 2020 hat diese Tendenz noch weiter verschärft.

In Kolumbien sind es nicht externe Akteure, sondern interne bzw. teilweise transnationale Gruppen, die das Gewaltgeschehen im Kontext der Umsetzung des Friedensabkommens und der Covid-19-Pandemie bestimmen. Da sich die Guerillagruppen seit den 1980er Jahren in hohem Maß über die illegale Ökonomie von Drogenproduktion und Drogenhandel finanzieren, ist die Unterscheidung zwischen politischen und kriminellen Akteuren unscharf und oft selbst in hohem Maße politisch (Duncan 2014). Bei den GegnerInnen des Friedensprozesses dominiert der Diskurs, die FARC-EP seien „Narco-Terroristen“, denen keine Zugeständnisse wie die im Friedensvertrag vereinbarte gesicherte Teilnahme in Parlament und Senat gemacht werden dürften. Fakt ist, dass auch nach der Unterzeichnung des Friedensvertrags 2016 die Gewalt vor allem in den abgelegenen, ländlichen Teilen des Landes weiterging. Die staatlichen Sicherheitskräfte haben sich als unfähig, teilweise auch als unwillig erwiesen, in den peripheren Regionen präsent zu sein, die von den FARC-EP im Zuge des Demobilisierungsprozesses verlassen wurden. Hier tobt seither ein Machtkampf um die territoriale und soziale Kontrolle. Daran haben auch die Pandemie und die damit verbundenen Einschränkungen der Mobilität nichts geändert.

Im Kontext der Pandemie ist die Gewalt – gemessen an den Morden – auf nationaler Ebene im Vergleich zum Vorjahr im März und April 2020 zunächst gesunken, was in den Regionen mit Präsenz des ELN dem einseitigen Waffenstillstand geschuldet sein dürfte. Im Mai und Juni stieg die Gewalt aber wieder deutlich an.^1^ Drei Konfliktlinien sind hier mit unterschiedlichen subnationalen Schwerpunkten bedeutsam (Llorente 2020): Erstens: Unterschiedliche Gewaltakteure (ELN, kriminelle Gruppen sowie Dissidenten der FARC) kämpfen um Einfluss und Kontrolle über die finanziell sehr lukrative Drogenproduktion und den Drogenhandel. Diese Auseinandersetzungen, vor allem in den Grenzregionen zum Amazonas und an der Pazifikküste, gehen unvermindert weiter. Obwohl Drogenproduktion und -handel durch die Pandemie erschwert werden, weil es beispielsweise keine Chemikalien zur Weiterverarbeitung von Coca-Blättern gibt, sind die kriminellen Netzwerke in hohem Maß flexibel und anpassungsfähig (UNODC 2020). Sie ändern entweder ihre Handelsrouten oder weichen in andere Geschäftsfelder aus. So mehren sich Berichte, dass diese Gruppen in den Amazonas vordringen und dort Tropenwald roden.

Zweitens: Mit dem Ende des Bürgerkrieges in Kolumbien wurde die Gewalt gegen MenschenrechtsverteidigerInnen und RepräsentantInnen zivilgesellschaftlicher Organisationen in wesentlich höherem Maße sichtbar als während des Krieges. Diese Gewalt sowohl gegen Einzelpersonen als auch in Form von Massakern geht trotz Pandemie-bedingtem Lockdown nicht nur unvermindert weiter, sondern hat sich sogar intensiviert. Sie betrifft die Akteure, die eine zentrale Rolle bei der zivilen Konflikttransformation haben, weil sie sich dafür einsetzen, dass strukturelle Konflikte, wie der Zugang zu Land oder die Rechte indigener Völker und der afro-kolumbianischen Bevölkerung, ohne Gewalt bearbeitet werden. Seit der Unterzeichnung des Friedensabkommens sind nach Angaben der Nichtregierungsorganisation INDEPAZ (2020) bis Mitte Juli 2020 971 MenschenrechtsverteidigerInnen ermordet worden. Die Morde konzentrieren sich vor allem in den Regionen Cauca, Nariño und Antioquia, in denen Drogenanbau oder extraktive Projekte, z. B. Goldabbau, auf Mobilisierung und Widerstand der lokalen Bevölkerung treffen.

Drittens: Insbesondere die Grenzregionen zu Venezuela, Panama und Ecuador, die Teil strategischer Korridore der illegalen Ökonomie sind, sind in hohem Maße gewaltsam. Auch hier überlagern sich die Aktivitäten verschiedener Gewaltakteure. Das ELN ist beispielsweise zunehmend nicht nur in Kolumbien präsent, sondern Bestandteil der wachsenden illegalen Ökonomie in Venezuela. Ähnliches gilt für die Teile der FARC, die sich nicht demobilisiert haben. Die bilateralen Beziehungen zwischen Kolumbien und Venezuela waren vor diesem Hintergrund in den vergangenen Jahren schlecht und von gegenseitigen Vorwürfen geprägt, bewaffnete Akteure zu unterstützen, die den Umsturz vorantreiben. Im Jahr 2019 wurden die diplomatischen Beziehungen abgebrochen, im Kontext der Pandemie die Grenzen einmal mehr geschlossen. Dies hindert nichtstaatliche Gewaltakteure aber nur wenig, ist die circa 2000 km lange Grenze doch schlecht zugänglich und kaum zu kontrollieren.

## Schlussfolgerungen: Covid-19 als Chance für den Frieden?

Der vorliegende Forumsbeitrag analysiert, wie die Covid-19-Pandemie die Muster von Gewalt und Frieden nach bzw. am Ende von Bürgerkriegen beeinflusst. Angesichts des Ausbruchs von Covid-19 in Kolumbien und Syrien im März 2020, den sich sehr dynamisch entwickelnden Auswirkungen des Virus sowie in Anbetracht der unsicheren Datenlage, vor allem in Syrien, müssen die Ergebnisse notwendigerweise vorläufig bleiben. Es zeigt sich jedoch, dass Covid-19 in den ersten Monaten offenbar bestehende Tendenzen hinsichtlich Gewalt und Frieden in Kolumbien und Syrien verstärkt. Dies deckt sich auch mit den früheren Erkenntnissen der Friedens- und Konfliktforschung, denen zu Folge friedensfördernde Effekte von Pandemien, wie die Kooperation zwischen Konfliktparteien, nur in sehr spezifischen Situationen und eher kurzfristig erwartet werden sollten.

Die Erfahrung mit den Waffenstillständen zeigt dies deutlich, offenbart jedoch auch interessante Unterschiede zwischen Kolumbien und Syrien: In Kolumbien sprach sich die ELN-Guerillagruppe aus einer Position der Schwäche heraus und mit Verweis auf UN-Generalsekretär Guterres zwar anfänglich für eine einseitige Waffenruhe im Zeichen der Pandemie aus, dieses „Angebot“ wurde jedoch von der Regierung Duque rundum abgelehnt, weil übergreifende Macht- und Sicherheitsinteressen der Staatsführung dominieren. Eine Annäherung oder gar Gespräche sind unter der Regierung Duque nicht zu erwarten. Auch in Syrien ist ein umfassender, gar landesweiter Waffenstillstand angesichts des sich abzeichnenden, brutalen „Siegfriedens“ zugunsten des Regimes und seiner externen Verbündeten denkbar unwahrscheinlich. Subnational ist es in der Region Idlib Anfang März 2020 jedoch zu einer fragilen Waffenruhe gekommen, die bis dato weithin fortbesteht. Anders als in Kolumbien sind es mit Russland und der Türkei in Syrien zwei externe Akteure, die als Garantiemächte der Waffenruhe fungieren. Covid-19 hat in diesem Zusammenhang eher begrenzt und indirekt für die Waffenruhe förderlich gewirkt.

Bei der Betrachtung der fragmentierten Territorialkontrolle und der Rolle (nicht‑)staatlicher Gewaltakteure in Syrien und Kolumbien hat Covid-19 die vor Ausbruch der Pandemie bestehenden Trends eher verschärft und somit erneute, lokale Eskalationen der Gewalt wahrscheinlicher gemacht. In Syriens Nordosten bestehen eine Vielzahl staatlicher wie nichtstaatlicher, syrischer wie nahöstlicher und außerregionaler Gewaltakteure. Hier hat sich die humanitäre Lage seit Beginn des Ausbruchs von Covid-19 deutlich verschlechtert, da die für die Versorgung der Region mit Lebensmitteln und Medikamenten notwendigen Grenzübergänge geschlossen sind und das hiervon profitierende Assad-Regime die internationalen Güter nicht in das verfeindete, stark kurdisch bzw. türkisch dominierte Gebiet weiterleitet. In Kolumbien bedeutet der Ausbruch der Pandemie, dass die Gewalt gegen die MenschenrechtsverteidigerInnen sichtbarer wird. Die vielfältigen, nichtstaatlichen Gewaltakteure, die im Drogenanbau und Drogenhandel ihre Haupteinnahmequellen haben, sind in Zeiten der Pandemie gezwungen, ihre illegalen ökonomischen Strategien anzupassen. Sie haben ihre soziale und territoriale Kontrolle mit negativen Konsequenzen für den Friedensprozess gestärkt. Die Regierung nutzt die Priorisierung der Pandemiekontrolle außerdem dazu, die Umsetzung des Friedensvertrages weiter zu unterminieren.

Allgemein gesprochen zeigen die bisherigen Erfahrungen aus Kolumbien und Syrien, dass Covid-19 vor Ort kurzfristig weniger als „game changer“ denn als Verstärker und Beschleuniger von Dynamiken wirkt, die bereits vor Ausbruch der Pandemie bestanden hatten.

## Footnotes

1 Daten aus Forensis Januar–Mai 2019 und 2020 https://www.medicinalegal.gov.co/cifras-estadisticas/boletines-estadisticos-mensuales bis Mai 2020; sowie der Polizei (Januar–Juni 2019 und 2020), die insgesamt höher sind, aber denselben Trend spiegeln https://www.policia.gov.co/delitos-de-impacto/homicidios.
